# Dietary diversity practice and its associated factors among pregnant women in Eastern Ethiopia: A community‐based cross‐sectional study

**DOI:** 10.1002/fsn3.3892

**Published:** 2023-12-11

**Authors:** Habtamu Geremew, Samuel Abdisa, Ebisa Zerihun, Yitagesu Kifelew Gizaw, Yoseph Kassa, Chalachew Gashu, Mulat Belay Simegn

**Affiliations:** ^1^ College of Health Science Oda Bultum University Chiro Ethiopia; ^2^ Department of Midwifery, College of Health Science Oda Bultum University Chiro Ethiopia; ^3^ Department of Nursing, College of Health Science Oda Bultum University Chiro Ethiopia; ^4^ Department of Statistics, College of Natural and Computational Science Oda Bultum University Chiro Ethiopia; ^5^ Department of Public Health, College of Medicine and Health Science Debre Markos University Debre Markos Ethiopia

**Keywords:** dietary diversity, Eastern Ethiopia, pregnant women

## Abstract

Micronutrient insufficiencies during pregnancy have a marked impact on the health of the woman and her offspring. Evidence about the dietary practice of pregnant women is limited in Ethiopia, particularly in drought‐prone areas where food insecurity is widely seen. Therefore, this research aimed to assess the dietary diversity practice and associated factors among pregnant women in Chiro district, Eastern Ethiopia. We employed a community‐based cross‐sectional survey in Chiro district, Eastern Ethiopia. The data were collected from 417 randomly selected pregnant women using an interview‐administered structured questionnaire. EpiData‐3.1 and STATA‐14 were used for data entry and analysis, respectively. The binary logistic regression analysis was deployed to assess the association between dietary diversity practice and predictor variables. Out of 420 calculated sample size, 417 pregnant women completed the survey giving a response rate of 99.3%. The overall prevalence of optimal dietary diversity was 38.4% (95% CI: 33.7%, 43.2%). Educational status (adjusted odds ratio [AOR]: 2.71, 95% CI: 1.08, 6.81), meal frequency (AOR: 1.91, 95% CI: 1.11, 3.28), home gardening (AOR: 4.21, 95% CI: 2.48, 7.16), and household food security (AOR: 0.23, 95% CI: 0.13, 0.40) were independent predictors of dietary diversity practice.This study found that a substantial proportion of pregnant women had suboptimal dietary diversity, indicating a fundamental micronutrient inadequacy. Educational status, meal frequency, home gardening practice, and household food security were independent determinants of dietary diversity practice. The findings suggest that promoting maternal education and home gardening practice, and controlling food insecurity might enhance optimal dietary diversity.

## Graphical abstract

This community‐based cross‐sectional study investigated the dietary diversity practice of pregnant women. Accordingly, the majority of women had poor dietary diversity. Hence calling for comprehensive intervention to prevent micronutrient deficiency among these special population.

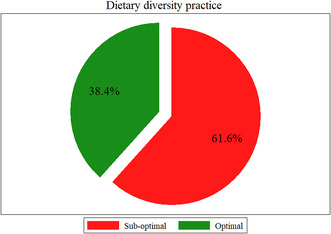

## 1 BACKGROUND

Dietary diversity is the measure of the range of meals eaten across and within food groups over a referenced period (FAO: Minimum dietary diversity for women: A guide for measurement, 2016). It is a qualitative index of dietary intake that can be used for evaluating the sufficiency of micronutrients (FAO: Minimum dietary diversity for women: A guide for measurement, 2016; Zhong et al., 2022). Intake of a diversified diet is crucial to satisfy the micronutrient requirement of pregnant women and promotes the physiologic development of the fetus (Tilahun & Kebede, 2021; Yang et al., 2021).

Although pregnant women have extra nutritional demand, poor dietary diversity practice among these vulnerable segments of the population is still a common problem worldwide; especially, in low and middle‐income countries (Hailu & Woldemichael, 2019; Lander et al., 2019). For instance, a community‐based study in Nepal found that about half of pregnant women did not consume a diverse diet (Shrestha et al., 2021). Another report from Ghana found that two out of every ten pregnant women were not consuming a diversified diet (Saaka et al., 2021). In Ethiopia, previous studies among pregnant women reported poor dietary diversity practices ranging from 38.8% to 87.2%, indicating a substantial variation across communities (Jemal & Awol, 2019; Workicho et al., 2019).

Micronutrient insufficiencies during pregnancy have a marked effect on the health of both the woman and her offspring (Yang et al., 2021, 2022). Low dietary diversity during pregnancy can result in hemorrhage, anemia, preterm birth, infection, and death in mothers (Zerfu et al., 2016). The fetus also suffers from various sequelae including stillbirth, low birth weight, congenital anomalies, and developmental delays (Abu‐Saad & Fraser, 2010; Saaka, 2012).

Dietary diversity practice is predicted by various factors. Existing evidence shows that dietary diversity practice has a significant association with educational status, family size, residence, household wealth index, household food security, antenatal care (ANC), home gardening, and meal frequency (Diddana, 2019; Hailu & Woldemichael, 2019; Saaka et al., 2021). Though, their level of importance differs across studies.

The National Nutrition Program is one of the strategies being implemented to overcome nutritional problems in Ethiopia, yet the problem continues to be substantial (Azene et al., 2021). Evidence about pregnant women's dietary diversity pattern is important to verify the extent and distribution of the phenomenon, to make informed decisions, and for efficient channeling of resources; however, such data are limited in Ethiopia, particularly in drought‐prone areas where food insecurity is widely seen. Therefore, this research aimed to assess dietary diversity practice and its associated factors among pregnant women in Chiro district, Eastern Ethiopia.

## 2 METHODS AND MATERIALS

### 2.1 Study design and setting

A community‐based cross‐sectional survey was carried out in Chiro district, West Hararghe Zone, Oromia Regional State, Ethiopia. The district is located 326 km east of the national capital, Addis Ababa. There are 42 (three urban and 39 rural) kebeles in the district with an estimated total population of 314,056 people, of which 10,898 are pregnant women (Geremew et al., 2023). The health service within the district is facilitated by 42 health posts, eight health centers, and one General Hospital, which are providing health‐promotive, disease‐preventive, curative, and rehabilitative health services including nutritional support. The study was conducted from November 1 to 30, 2022.

### 2.2 Population

All pregnant women in Chiro district was our source population. Whereas, all pregnant women in the randomly selected kebels of Chiro district constitute our study population.

### 2.3 Eligibility criteria

All pregnant women who are living in Chiro district for at least 6 months were eligible to be included in this study. Nevertheless, pregnant women who were seriously ill and/or have difficulties to communicate were excluded from this survey.

### 2.4 Sample size and sampling procedures

The single population proportion formula was used to calculate the required sample size by considering 45% dietary diversity practice from a community‐based study in East Gojjam Zone (Yeneabat et al., 2019), 95% confidence interval, and a 5% margin of error. As a result, the estimated sample size was 381. An anticipated 10% non‐response rate was added giving us a final sample size of 420 pregnant women.

Systematic random sampling technique was employed to select study participants from eight randomly selected kebeles, by using the pregnancy screening registration book of health posts as a sampling frame. Besides, the sample size was distributed to each selected kebeles proportionally (proportional to the number of pregnant women in each kebeles).

### 2.5 Data collection and quality assurance

An interview‐administered structured questionnaire that was adapted by reviewing different literatures was utilized to gather the data (Boke & Geremew, 2018; Tilahun & Kebede, 2021; Yeneabat et al., 2019). The tool was first prepared in English, and then translated to Oromiffa and back to English by two different language experts to ensure its consistency. Eight clinical nurses who are working in primary health care facilities, and have extensive experience in human nutrition were recruited as data collectors and they were supervised by four B.Sc. nurses.

To ensure the quality of the collected data, data collectors and supervisors were trained for 2 days about the study, and how to approach and interview pregnant women. The questionnaire was also pretested on 30 pregnant women in one of the kebeles not included in this survey. Furthermore, the quality and completeness of the collected data were reviewed daily by investigators.

### 2.6 Operational definition and study variables

#### 2.6.1 Dependent variable

Dietary diversity was our dependent variable. Pregnant women who had consumed five or more out of the 10 food groups in the past 24 h were considered to practice optimal dietary diversity (FAO: Minimum dietary diversity for women: A guide for measurement, 2016).

#### 2.6.2 Independent variables

The independent variable considered in this study includes socio‐demographic variables, housing and environmental variables, and reproductive and behavioral variables. Besides, household food security was assessed using the Household Food Insecurity Access Scale (HFIAS). The tool is composed of nine items specific to an experience of food insecurity occurring within the previous 4 weeks; accordingly, a household was categorized as food‐secure if it does not experience any of the food insecurity conditions or just experienced worry, but rarely (Coates et al., 2007).

### 2.7 Data processing and analysis

The data were entered into the computer system using EpiData‐3.1, and then it was exported to STATA‐14 statistical software for further statistical analysis. Descriptive analysis was performed and presented in tables and figures. Binary logistic regression was fitted to identify predictors of dietary diversity practice. The bi‐variable logistic regression model was built to select candidate variables for the multivariable analysis. Then independent variables with *p*‐value less than .25 were entered into the multivariable logistic regression model, after assessing for multi‐collinearity. *p*‐values less than .05 was used to declare a statistically significant association. Besides, the best model was selected using the likelihood ratio test, and the fitness of the final model was checked using the Hosmer–Lemeshow goodness‐of‐fit test.

## 3 RESULTS

### 3.1 Socio‐demographic characteristics

Out of 420 randomly selected pregnant women, 417 women completed the survey, yielding a response rate of 99.3%. The mean age of the respondents was 26.86 (SD ± 6.12) years. The majority (84.4%) of pregnant women were rural dwellers and most (66.2%) of the study participants were housewives (Table 1).

**TABLE 1:** Distribution of sociodemographic characteristics among pregnant women in Chiro district, Eastern Ethiopia, 2022.

Variables	Category	Frequency	Percentage
Age group	15–24 years	169	40.5
	25–34 years	186	44.6
	35–45 years	62	14.9
Residence	Urban	65	15.6
	Rural	352	84.4
Marital status	Married	404	96.9
	Divorced	10	2.4
	Widowed	3	0.7
Educational status	No formal education	175	42.0
	Primary	208	49.9
	Secondary and above	34	8.1
Occupation	Housewife	276	66.2
	Farmer	110	26.4
	Merchant	15	3.6
	Government employee	13	3.1
	Daily laborer	3	0.7
Family size	≤4	210	50.4
	>4	207	49.6
Income category	Highest	85	20.4
	Mild	92	22.1
	Moderate	87	20.8
	Lowest	153	36.7
Time to reach the market	≤30 min	162	38.8
	30–60 min	170	40.8
	>60 min	85	20.4

### 3.2 Housing and environmental characteristics of study participants

The majority of pregnant women were from households that own latrines (82.0%), large livestock (59.5%), small livestock (68.6%), and produce crops (81.3%). The food source of more than half (53.7%) of women was their own production (Table 2).

**TABLE 2:** Distribution of housing and environmental characteristics among pregnant women in Chiro district, Eastern Ethiopia, 2022.

Variable	Categories	Frequency	Percentage
Home gardening	Yes	180	43.2
	No	237	56.8
Production of crops	Yes	339	81.3
	No	78	18.7
Having large live‐stocks	Yes	248	59.5
	No	169	40.5
Having small live‐stock	Yes	286	68.6
	No	131	31.4
Does the household own latrine	Yes	342	82.0
	No	75	18.0
Source of drinking water	Pipe	144	34.5
	Spring	226	54.2
	River	47	11.3
Food source	Own production	224	53.7
	Purchased	80	19.2
	Both	113	27.1
Household food security	Food secure	256	61.4
	Not food secure	161	38.6

### 3.3 Reproductive and behavioral characteristics of respondents

Most (91.4%) of pregnant women were not in a polygamous relationship. About three out of 10 pregnancies were not planned. Most (52.5%) of the women were in their second trimester, and about one‐third of women receive support from their partner on household activities (Table 3).

**TABLE 3:** Distribution of reproductive and behavioral characteristics among pregnant women in Chiro district, Eastern Ethiopia, 2022.

Variable	Categories	Frequency	Percentage
Polygamy	Yes	36	8.6
	No	381	91.4
Gravidity	≤2	139	33.3
	3–4	144	34.5
	≥5	134	32.2
ANC follow‐up	Yes	305	73.1
	No	112	26.9
Nutrition information	Yes	301	72.2
	No	116	27.8
Is this pregnancy planned	Yes	291	69.8
	No	126	30.2
Stage of pregnancy	First trimester	61	14.6
	Second trimester	219	52.5
	Third trimester	137	32.9
Illness within 2 weeks	Yes	101	24.2
	No	316	75.8
Food taboo	Yes	30	7.2
	No	387	92.8
Meal frequency	<4	282	67.6
	≥4	135	32.4
Fasting during pregnancy	Yes	269	64.5
	No	148	35.5
Khat chewing	Yes	267	64.0
	No	150	36.0
Partner support on indoor activities	Yes	139	33.3
	No	278	66.7

### 3.4 Prevalence of dietary diversity practice

**FIGURE 1:**
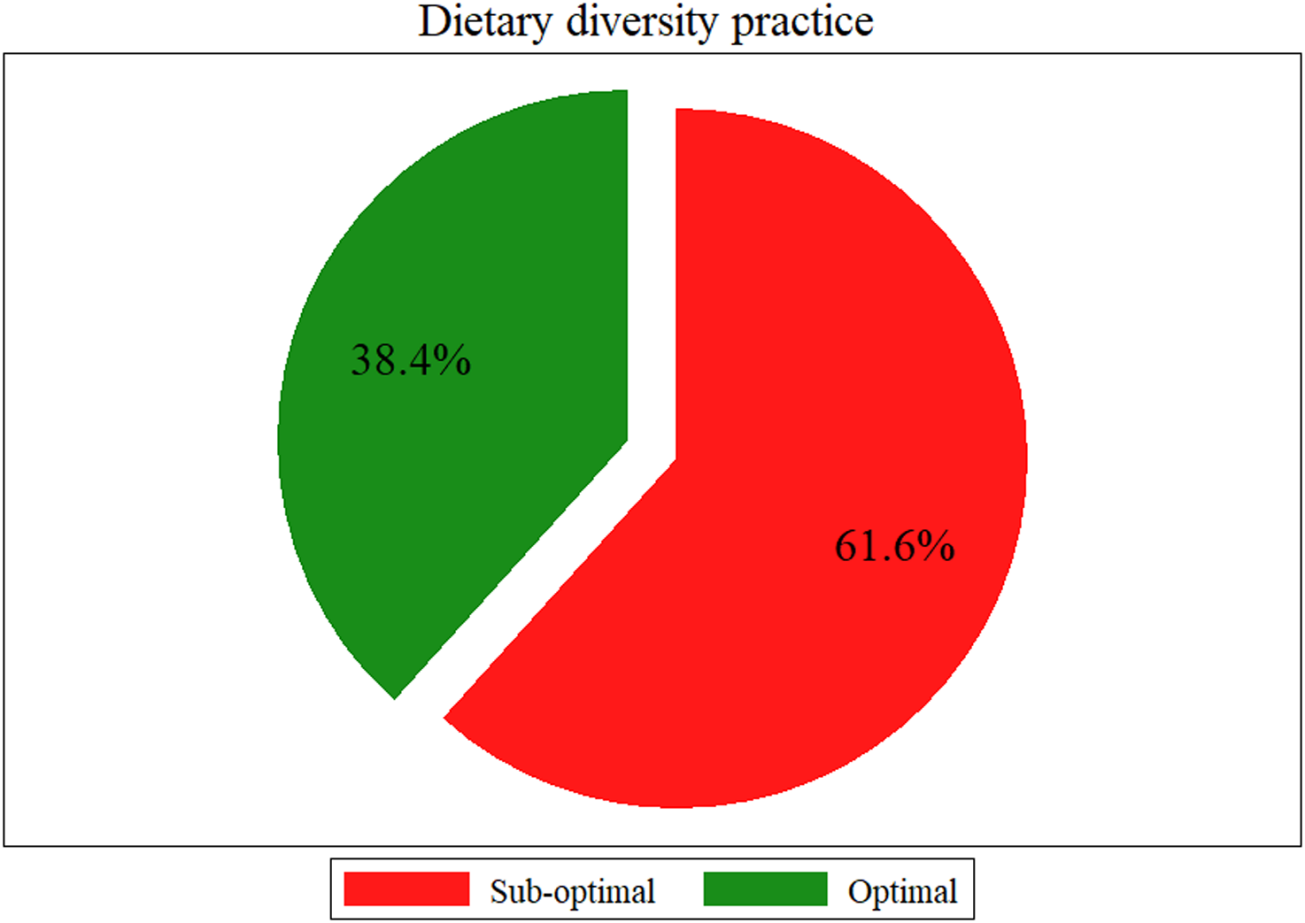
Dietary diversity practice of pregnant women in Chiro district, Eastern Ethiopia.

**FIGURE 2:**
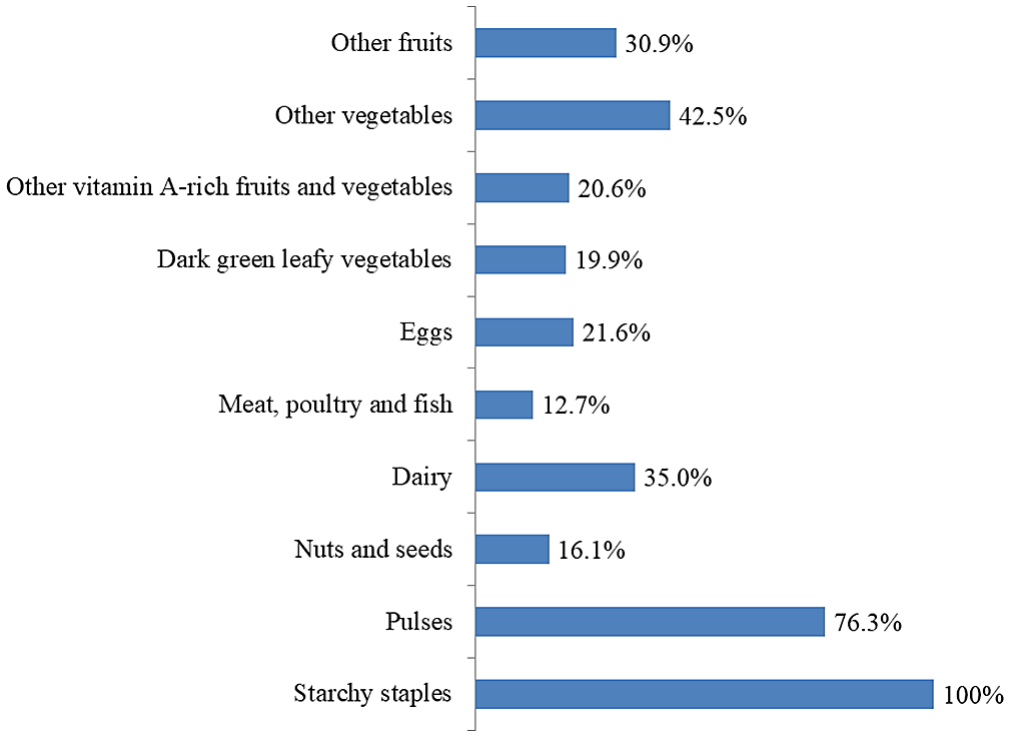
Food groups consumed in the past 24 h among pregnant women in Chiro district, Eastern Ethiopia.

Out of 417 respondents interviewed, 160 consumed a diversified diet in the past 24 h, indicating an optimal dietary diversity prevalence of 38.4% (95% CI: 33.7%, 43.2%) (Figure 1). The most commonly consumed food groups were starchy staples (100%), followed by pulses (76.3%) and other vegetables (42.5%) (Figure 2).

### 3.5 Determinants of dietary diversity practice

According to the multivariable logistic regression analysis, educational status, meal frequency, household food security status, and home gardening practice were significant determinants of dietary diversity practice.

Correspondingly, pregnant women who had attended secondary or higher education were 2.71 (95% CI: 1.08, 6.81) times more likely to consume an optimally diverse diet than women who attended only primary education. Similarly, pregnant women who had four or more meals in a day were 1.91 (95% CI: 1.11, 3.28) times more likely to consume a diversified diet than women who had less than four meals in a day. The odds of optimal dietary diversity were 4.21 (95% CI: 2.48, 7.16) times more common among women who are from households that practice home gardening as compared to their counterparts. Pregnant women who are from food‐insecure households were 0.23 (95% CI: 0.13, 0.40) times less likely to practice optimal dietary diversity than women from food‐secure households (Table 4).

**TABLE 4:** Factors associated with dietary diversity practice of pregnant women in Chiro district, Eastern Ethiopia, 2022.

Variable	Categories	Dietary diversity		COR	AOR (95% CI)
		Optimal	Sub‐optimal		
Occupation	Housewife	101 (36.6)	175 (63.4)	1	1
	Farmer	42 (38.2)	68 (61.8)	1.07	0.66 (0.38, 1.17)
	Others	17 (54.8)	14 (45.2)	2.10	1.09 (0.42, 2.79)
Family size	≤4	91 (43.3)	119 (56.7)	1	1
	>4	69 (33.3)	138 (66.7)	0.65	0.74 (0.45, 1.23)
Educational status	No formal education	55 (31.4)	120 (68.6)	0.72	1.12 (0.66, 1.90)
	Primary	81 (38.9)	127 (61.1)	1	1
	Secondary & above	24 (70.6)	10 (29.4)	3.76	2.71 (1.08, 6.81)*
Illness in the past 2 weeks	Yes	27 (26.7)	74 (73.3)	0.50	0.76 (0.42, 1.37)
	No	133 (42.1)	183 (57.9)	1	1
Nutrition information	Yes	137 (45.5)	164 (54.5)	1	1
	No	23 (19.8)	93 (80.2)	0.30	0.61 (0.33, 1.14)
Meal frequency	<4	78 (27.7)	204 (72.3)	1	1
	≥4	82 (60.74)	53 (39.3)	4.05	1.91 (1.11, 3.28)*
Kchat chewing	Yes	86 (32.2)	181 (67.8)	1	1
	No	74 (49.3)	76 (50.7)	2.05	1.29 (0.78, 2.15)
Home gardening	Yes	95 (52.8)	85 (47.2)	2.96	4.21 (2.48, 7.16)*
	No	65 (27.4)	172 (72.6)	1	1
Food security	Food secure	132 (51.6)	124 (48.4)	1	1
	Not food secure	28 (17.4)	133 (82.6)	0.20	0.23 (0.13, 0.40)*

Abbreviations: AOR, adjusted odds ratio; COR, crude odds ratio.

* Significant at *p* < .05.

## 4 DISCUSSION

In this cross‐sectional analysis, we investigated the dietary diversity practice of pregnant women in Chiro district, Eastern Ethiopia. The results showed that 38.4% of pregnant women in the study area had optimal dietary diversity practices, which is in line with a community‐based study in southern Ethiopia, where the magnitude of optimal dietary diversity was 42.1% (Gudeta et al., 2022). However, our finding was higher than another report from Gurage Zone, southern Ethiopia (Geta et al., 2022). The plausible explanation for this variation might due to the difference in methodology, which the study conducted in the Gurage zone was based on repeated measurements during different gestational ages of pregnancy.

On the other hand, our finding was lower than reports of previous studies conducted in Harar town, Eastern Ethiopia, 52.7% (Kassahun et al., 2023); Jeldu District, Central Ethiopia, 81.9% (Merga et al., 2022); Bale zone southeast Ethiopia, 45.8% (Hailu & Woldemichael, 2019); and Kaffa Zone, southwest Ethiopia, 51% (Tilahun & Kebede, 2021). This discrepancy might be due to the differences in study setting and study period across the researches. The present study was conducted in Chiro district, known for high and rapidly shifting production of khat (Tofu & Wolka, 2023). This may cause poor access and availability of different food groups like fruits and vegetables in the area, thus leading to minimal dietary diversity.

In the present study, pregnant women who had secondary or higher education had a higher chance of optimal dietary diversity as compared to women with primary or lower education. This finding is congruent with previous reports from Ethiopia (Geta et al., 2022), and Kenya (Kiboi et al., 2017), and could be due to the greater probability of acquiring important information about proper feeding among women with higher educational levels. Hence, acknowledging the comprehensive role of education on health.

In line with previous studies (Jemal & Awol, 2019; Yeneabat et al., 2019), pregnant women who feed four or more times had a higher chance of eating a diversified diet than women who feed less than four times in a day. This might be due to the higher chance of consuming different food groups with increasing meal frequencies. Similarly, the likelihood of optimal dietary diversity was higher among pregnant women who are from households with home gardening as compared to their counterparts. This might be due to the expanded availability of different food groups among women from home gardening households (Blakstad et al., 2021).

This study found that pregnant women from food‐secure households had higher odds of optimal dietary diversity than women from food‐insecure households. The finding is compatible with prior evidence from Ethiopia (Geta et al., 2022; Jemal & Awol, 2019), and Bangladesh (Na et al., 2016). The possible explanation could be due to the lack of access to enough food among food‐insecure households thus compromising diversity (quality) over quantity.

This study has certain limitations. First, the cross‐sectional study design hinders any causal inferences between dietary diversity and its correlates. Second, the effect of seasonal variation on dietary diversity practice is not considered in this analysis. Finally, relying on women's memorization of food groups consumed might introduce recall bias.

## 5 CONCLUSION

This study indicates that the majority of pregnant women in the study area had suboptimal dietary diversity, indicating a fundamental micronutrient inadequacy. Educational status, meal frequency, home gardening practice, and household food security were independent determinants of dietary diversity practice. The findings suggest that promoting maternal education and home gardening practice, and controlling food insecurity might enhance optimal dietary diversity.

## AUTHOR CONTRIBUTIONS

**Habtamu Geremew:** Conceptualization (lead); data curation (lead); formal analysis (lead); funding acquisition (lead); investigation (lead); methodology (lead); project administration (lead); resources (equal); software (lead); supervision (lead); validation (lead); visualization (lead); writing – original draft (lead); writing – review and editing (lead). **Samuel Abdisa:** Data curation (equal); funding acquisition (equal); investigation (equal); methodology (equal); resources (equal); writing – review and editing (equal). **Ebisa Zerihun:** Investigation (equal); methodology (equal); project administration (equal); validation (equal); visualization (equal); writing – review and editing (equal). **Yitagesu Kifelew Gizaw:** Data curation (supporting); formal analysis (equal); investigation (equal); methodology (equal); software (equal); supervision (equal); validation (equal); writing – review and editing (equal). **Yoseph Kassa:** Data curation (equal); investigation (equal); methodology (equal); validation (equal); writing – review and editing (equal). **Chalachew Gashu:** Investigation (equal); methodology (equal); project administration (equal); supervision (equal); writing – review and editing (equal). **Mulat Belay Simegn:** Formal analysis (equal); investigation (equal); methodology (equal); project administration (equal); validation (equal); writing – review and editing (equal).

## FUNDING INFORMATION

This research was funded by Oda Bultum University. The funders had no role in the study design, data collection, analysis and interpretation, or preparation of the manuscript.

## CONFLICT OF INTEREST STATEMENT

The authors declare no competing interests in this work.

## ETHICS STATEMENT

This study was approved by Oda Bultum University ethical review committee. The Declaration of Helsinki principles were strictly followed during the conduct of this study. due to the non‐invasive nature of the data collection process, oral informed consent was obtained from all pregnant women who participated in this study. Besides, the confidentiality of the collected information was assured by not recording women's identifiers.

## Data Availability

The datasets used in this study can be obtained from the corresponding author upon reasonable request.
